# Charles Fayette Taylor, M. D., 1827–1399

**Published:** 1899-05

**Authors:** L. A. Weigel


### OBITUARY.

### CHARLES FAYETTE TAYLOR, M. D., 1827-1899.

The death of Dr. Charles Fayette Taylor, of New York, ends the career of an American physician, to whom the whole world is indebted for his original and valuable work in orthopedic surgery.

Dr. Taylor was born in Williston, Vt., April 25, 1827, and was educated in the schools of his native town. After a short experience in teaching school, he decided to study medicine and was graduated from the Medical Department of the University of Vermont. After a few years spent in general practice, he began to turn his attention more especially to the treatment of functional neuroses and the correction of deformities. While he did much original work in the former class of cases it was his labors in the scientific advancement of orthopedy, that gave him the reputation he so well deserved. His great ingenuity, skill and wonderful attention to detail in the construction and use of mechanical appliances, were no doubt prominent factors in his successful treatment of deformities. An orthopedic appliance in his hands was an obedient servant directed by a master mind.

The establishment of the New York Orthopedic Dispensary and Hospital was due entirely to Dr. Taylor's efforts. In 1866, after having given a large share of his time and means to the free treatment of the crippled and deformed poor, at his private office, he called the attention of Howard Potter, Theodore Roosevelt and others to the necessity of better facilities for the care of these cases, and the New York Dispensary was founded. Its first home consisted of a single room in a building on Broadway, between 35th and 36th streets. In 1873 it was moved to the first of the buildings now occupied in 59th street. Here Dr. Taylor was the active surgeon for a period of eight years, when failing health compelled him to resign. For many years he also served as orthopedic surgeon to St. Luke's Hospital.

Although a man of ceaseless activity in his chosen field of labor, he found much to interest him outside of it, and never lost an opportunity to learn something from every one with whom he came in contact. His writings give evidence of that great originality of thought. He never wrote without having something to say and hence his works were of special value.

The many sterling qualities of Dr. Taylor's mind has left its impress on his followers and upon the whole profession. His thoroughness, his energy and skill, his kindness of heart and his sincerity and frankness will ever be remembered by those who were fortunate enough to come under his care as patients, and by those who knew him as a true friend and professional counselor, who could always be relied upon.

Dr. Taylor was honored with medals and diplomas at Paris in 1867, in Vienna in 1873 and at Philadelphia in 1876. During his active life he wrote many articles and books, of which The Theory and Practice of the Movement Cure, The Mechanical Treatment of Potts's Disease of the Spine and The Mechanical Treatment of Hip-joint Disease are the best known and may be regarded as classics.

L. A. WEIGEL.

